# A new parasitoid of *Bazaria turensis* (Lepidoptera, Pyralidae): *Campoplex bazariae* sp. n. (Hymenoptera, Ichneumonidae)

**DOI:** 10.3897/zookeys.466.8618

**Published:** 2014-12-18

**Authors:** Yu-Xiang Zhao, Mao-Ling Sheng

**Affiliations:** 1 The Key Laboratory for Silviculture and Conservation of Ministry of Education, Beijing Forestry University, Beijing 100083, P. R. China; 2 General Station of Forest Pest Management, State Forestry Administration, Shenyang 110034, P. R. China

**Keywords:** Campopleginae, new species, taxonomy, host, *Bazaria turensis*, Lepidoptera, host plant

## Abstract

A new solitary endoparasitoid of the larva of *Bazaria turensis* Ragonot, 1887 (Lepidoptera, Pyralidae) in Qinghai province, China, *Campoplex bazariae* Sheng, **sp. n.**, belonging to the subfamily Campopleginae (Hymenoptera, Ichneumonidae), is reported. Illustrations of the new species are provided.

## Introduction

*Campoplex* Gravenhorst, 1829, belonging to the subfamily Campopleginae (Hymenoptera: Ichneumonidae), comprises 209 species (Yu et al. 2012), of which 15 are from the Eastern Palaearctic Region (Momoi 1977, Uchida 1932, 1936, 1956, Yu et al. 2012), 123 from the Western Palaearctic (six of them are found across the Palaearctic) (Horstmann 1985, 1993, 2008, Meyer 1935, Yu et al. 2012), 33 are from the Nearctic Region (Yu et al. 2012), 30 from the Oriental (Gupta and Maheshwary 1977), 11 from the Neotropical, two from the Afrotropical (Townes and Townes 1973). Eleven species of *Campoplex* Gravenhorst have been known from China (Gupta and Maheshwary 1977, Kokujev 1915, Sheng and Sun 2014, Sonan 1930, Uchida 1932). The diagnostic characters of the genus were elucidated by Townes (1970) and expanded upon by Gupta and Maheshwary (1977).

The hosts of *Campoplex* Gravenhorst mainly belong to Coleophoridae, Gelechiidae, Pyralidae, Tortricidae, Yponomeutidae, etc. (Aubert 1983, Horstmann 1980, 1985, Kusigemati 1987, Shaw and Aeschlimann 1994, Yu et al. 2012).

In the last five years the authors have been exploring Qinghai Province, Ningxia Hui Autonomous Region and Inner Mongolia Autonomous Region, situated in northwestern China, and have collected large numbers of ichneumonids. In this article, one new species of *Campoplex* is reported, reared from the larva of *Bazaria turensis* Ragonot, 1887 (Lepidoptera, Pyralidae), from Qinghai Province, P.R. China.

## Materials and methods

Mature larvae of the host, *Bazaria turensis*, were collected on 28 August 2013 by Mao-Ling Sheng. Cocoons of the host were collected on 21 May 2014 by Yan-Ling Zhang, from a forest where there had been an outbreak lasting at least three years, and brought to the laboratory. The forest is located in Dulan County, Qinghai Province. The forest is a shrubbery composed of *Nitraria tangutorum* Bobrov, Lycium chinense Miller var. potaninii (Pojarkova) A.M. Lu and *Kalidium foliatum* (Pallas) Moquin-Tandon. Mature larvae were maintained in a nylon cage at room temperature. The pupae were stored individually in glass tubes with a piece of filter paper dipped in distilled water to maintain moisture and plugged tightly with absorbent cotton. Glass tubes are 60 mm long and 6 mm diameter. After the emergence of moths and parasitoids was complete, all remaining pupae were dissected to record their condition (i.e. status of moths, and parasitism).

Specimens were compared with material from the Natural History Museum (NHM), London, UK. Morphological terminology is mostly based on Gauld (1991).

Images of whole insects were taken using a CANON Power Shot A650 IS. Other images were taken using a Cool SNAP MPS Color attached to a Zeiss Discovery V8 Stereomicroscope and captured with QCapture Pro 7.

Type specimens are deposited in the Insect Museum, General Station of Forest Pest Management (GSFPM), State Forestry Administration, People’s Republic of China.

## Results

### Campoplex

Taxon classificationAnimaliaHymenopteraIchneumonidae

Gravenhorst, 1829

Campoplex Gravenhorst, 1829. Ichneumonologia Europaea, 3: 453. Type-species: *Ichneumon difformis* Gmelin, 1790. Designated by Westwood 1840.

#### Diagnosis.

Eye slightly or not at all emarginate opposite antennal socket. Occipital carina joining hypostomal carina above base of mandible, or reaching directly to base of mandible. Area superomedia and area petiolaris confluent, junction between them usually discernible, combined area moderately wide. Area dentipara completely bordered by carinae. Apex of propodeum usually not reaching middle of hind coxa. Areolet usually present. 2m-cu inclivous. Basal portion of first tergite subcylindric and less than 3.0× as long as deep, suture between tergite and sternite approximately at or a little below mid-height. Apex of male gonosquama rounded above or with a very shallow emargination. Ovipositor sheath about 3–4× as long as apical depth of metasoma.

### Campoplex bazariae

Taxon classificationAnimaliaHymenopteraIchneumonidae

Sheng sp. n.

http://zoobank.org/FFA02389-23EE-4CD5-9A3B-02734A01D902

Figs 1–5 , 6–10

#### Etymology.

The specific name is derived from the host’s name.

#### Material examined.

Holotype, female emerged from cocoon of *Bazaria turensis* on 20 July 2014 reared by Yan-Ling Zhang, CHINA: Balong, 2860m, Dulan County, Qinghai Province. Paratypes: 2 females, same data as holotype. 1 male, same data as holotype except 15 September 2014. 1 female, 1 male, CHINA: Nuomuhong, 2690m, Dulan County, Qinghai Province, 28 August 2013, Mao-Ling Sheng.

#### Diagnosis.

Face finely coriaceous, with dense punctures. Interocellar area with distinct punctures. Postocellar line 1.6–1.7× as long as ocular-ocellar line. Postscutellum with fine dense distinct punctures. First tergite from base to apex strongly evenly convex, smooth, shiny. Second and subsequent tergites finely coriaceous. Apical margins of tergites 6 and 7 with deep median triangular emarginations. Ovipositor slightly, evenly curved upwards. Head except mandibles and maxillary and labial palpi, mesosoma and all tergites entirely black.

#### Description.

Female. Body length 7.5–8.0 mm. Fore wing length 5.5–5.8 mm. Ovipositor sheath length 2.7–2.9 mm.

**Head.** Inner margins of eyes slightly convergent ventrally. Narrowest width of face (Fig. 2) approximately 0.9× height of face plus clypeus, slightly convex, finely coriaceous, with dense punctures. Clypeus shiny, with sparse punctures; apical margin slightly elevated and arched forwards. Mandible short, with large punctures, upper tooth as long as lower tooth. Malar area slightly concave, indistinctly granulose. Malar space approximately 0.30–0.34× as long as basal width of mandible. Gena in dorsal view approximately 0.6× as long as width of eye, almost smooth, with sparse, fine punctures, posterior portion obviously convergent posteriorly. Vertex (Fig. 3) finely granulose, with indistinct, fine, shallow punctures. Interocellar area with distinct punctures. Postocellar line 1.6–1.7× as long as ocular-ocellar line. Ocular-ocellar line 1.0–1.2× diameter of posterior ocellus. Frons almost flat, rough, with dense, indistinct punctures. Antenna with 37 flagellomeres. Ratio of length from first to fifth flagellomeres: 4.0:3.0:2.9:2.8:2.6. Occipital carina complete, upper median portion evenly up-curved, lower end reaching base of mandible.

**Figures 1–5.**
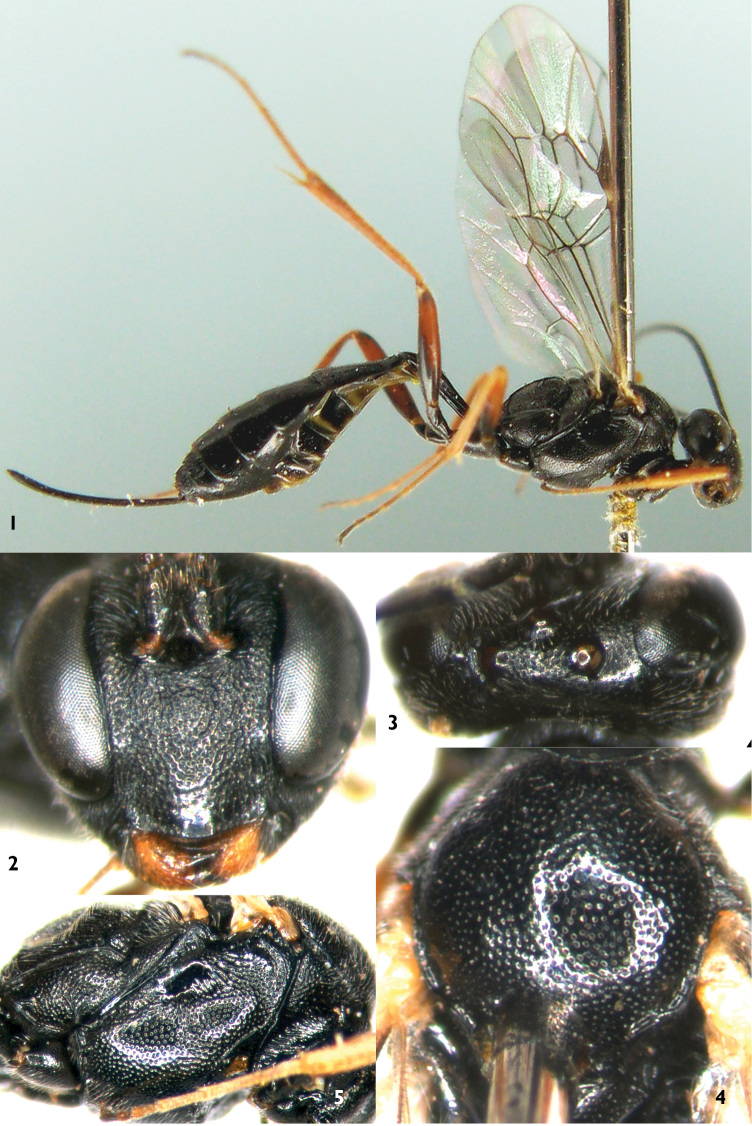
*Campoplex bazariae* sp. n. Holotype. Female **1** Habitus, lateral view **2** Head, anterior view **3** Head, dorsal view **4** Mesoscutum **5** Mesopleuron.

**Figures 6–10.**
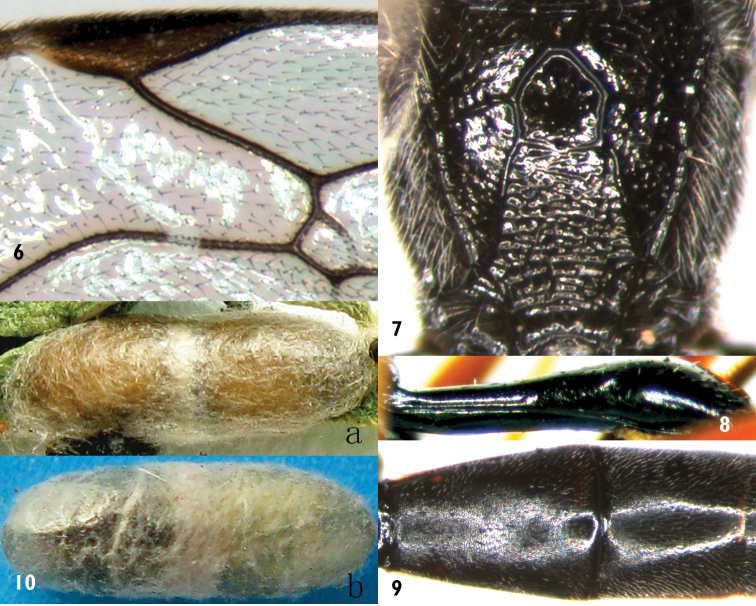
*Campoplex bazariae* Sheng, sp. n. Holotype. Female **6** Areolet and pterostigma **7** Propodeum **8** First tergite, lateral view **9** Tergites 2–3 **10 a, b** Cocoon.

**Mesosoma.** Lateral concavity of pronotum with dense oblique wrinkles, upper-posterior portion with dense coarse irregular punctures, distance between punctures 0.2–0.5× diameter of puncture, upper posterior margin with dense fine punctures. Epomia distinct. Mesoscutum (Fig. 4) evenly convex, with distinct punctures, distance between punctures 0.2–2.5× diameter of puncture. Notaulus vestigial. Scutellum evenly, strongly convex, with dense distinct punctures, distance between punctures 0.2–0.5× diameter of puncture. Postscutellum trapezoidally convex, with fine, dense, distinct punctures, anteriorly transversely concave. Mesopleuron (Fig. 5) with distinct punctures, distance between punctures approximately 0.2–2.5× diameter of puncture, in lower-front portion of speculum with dense oblique wrinkles. Speculum approximately transverse-quadrate, smooth, shiny. Upper end of epicnemial carina reaching about 0.5 level of posterior margin of pronotum. Mesopleural fovea consisting of short, shallow horizontal groove. Mesosternum with punctures as that of mesopleuron, posterior transverse carina complete, strong. Metapleuron slightly convex, with punctures as, or slightly denser than that of mesopleuron. Submetapleural carina complete, strong. Wings slightly brownish, hyaline. Fore wing with vein 1cu-a distinctly distal of 1-M. Areolet (Fig. 6) obliquely quadrangular, its petiole 0.7–0.9× as long as 2rs-m, receiving vein 2m-cu approximately 0.7× distance from vein 2rs-m to 3rs-m. 2m-cu slightly inclivous. 2-Cu approximately as long as 2cu-a. Hind wing vein 1-cu almost vertical, about 3.0× as long as cu-a. Ratio of lengths of hind femur, tibia and tarsus 7.5:10:12.5. Ratio of length of hind tarsomeres 1:2:3:4:5 is 10.0:4.0:2.6:1.7:2.0. Claws thin. Base of fore claw with sparse pectination. Base of hind claw with dense pectination. Area spiracularis of propodeum (Fig. 7) combined with area lateralis. Areas basalis small, strongly convergent posteriorly, longer than its maximum width, smooth, shiny. Area superomedia and area petiolaris confluent, junction point between them discernible. Area superomedia smooth, shiny, costula connecting approximately at its middle or slightly behind middle. Area petiolaris almost flat (indistinctly longitudinally concave), with dense distinct transverse wrinkles. Area externa smooth, distinctly punctate. Area dentipara slightly coarse, with indistinct, irregular wrinkles. Area posteroexterna with oblique transverse wrinkles. Areas spiracularis and lateralis with dense indistinct fine punctures. Propodeal spiracle small, elongate-oval, connecting with pleural carina by a distinct carina, space between them shorter than its longest diameter, distance to lateral longitudinal carina longer than its longest diameter. Apex of propodeum reaching 0.25 of hind coxa.

**Metasoma.** First tergite (Fig. 8) approximately 2.9 times as long as apical width, basal portion subcylindric, suture separating from sternite lying at mid height of segment; from base to apex strongly, evenly convex; smooth, shiny. Spiracle located about at apical 0.4 of first tergite. Second and subsequent tergites finely coriaceous. Second tergite (Fig. 9) 1.25–1.43× as long as apical width. Third and following tergites compressed. Apical margins of tergites 6 and 7 with deep median triangular emarginations. Ovipositor sheath approximately 1.25× as long as hind tibia, 0.65–0.75× as long as total length of posterior seven tergites. Ovipositor slightly curved upwards, with distinct subapical dorsal notch.

**Color** (Fig. 1). Black, except the following. Maxillary and labial palpi blackish brown. Median portions of mandibles dark brown, or upper-median margins yellowish brown. Tegula stramineous. All coxae and trochanters, except brownish apical margins of fore trochanter, black. Fore femur, dorsal profiles and ventral apical portions of mid and hind femora reddish brown. Basal ventral halves or more of mid and hind femora, apical portion of hind femur black. Fore and mid tibiae, except outsides slightly yellowish, and tarsi brown to dark brown. Ventral side of hind tibia reddish brown, dorsal side and tarsus dark brown. Second, lateral margin of third and apical margins of fourth to sixth sternites grayish yellow to off-white. Median portion of pterostigma dark brown. Veins brownish black.

**Male.** Body length 8.0–8.2 mm. Fore wing length approximately 6.0 mm. Median portion of frons with dense transverse wrinkles. Apex of gonosquama more or less horny. Median portion of mandible reddish brown. Tegula yellow, median portion asymmetrically blackish brown. Mid and hind tarsi dark brown.

**Cocoon** (Fig. 10). Length about 7.5 mm. Diameter about 2.5 mm. Apices vaulted. Whitish grey.

#### Host.

*Bazaria turensis* Ragonot, 1887 (Lepidoptera, Pyralidae).

#### Host plants.

*Nitraria tangutorum* Bobrov (Zygophyllaceae), *Kalidium foliatum* (Pallas) Moquin-Tandon (Amaranthaceae).

#### Biology.

*Campoplex bazariae* Sheng is a solitary endoparasitoid of the larva of *Bazaria turensis*, spinning its cocoon in deciduous leaves (Fig. 10a) or near the surface of soil (Fig. 10b); also in the cocoon of *Bazaria turensis*, collected and reared by the local colleague, Yan-Ling Zhang (Director of Forestry Pest Control and Quarantine Station of Dulan, Qinghai, China).

#### Remarks.

**Figures 11–12.**
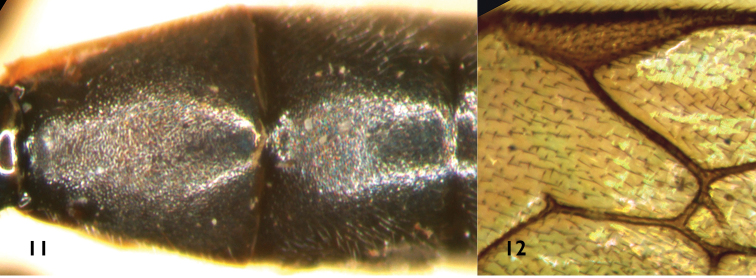
*Campoplex ovatus* (Brischke, 1880) (NHM) Female **11** Tergites 2–3 **12** Areolet and pterostigma.

This new species is similar to *Campoplex ovatus* (Brischke, 1880) and can be distinguished from the latter by the following combination of characters: petiole of areolet (Fig. 6) 0.7–0.9× as long as 2rs-m; area superomedia smooth, shiny, flat, costula connecting at its middle; area petiolaris almost flat; second tergite approximately 1.25–1.43× as long as apical width; posterior portions of tergites 6 and 7 with deep median triangular emarginations; apical portions and basal ventral halves or more of hind femora black; ventral profiles of hind tibiae reddish brown, dorsal profiles darkish brown; median portion of pterostigma darkish brown. *Campoplex ovatus* (Figs 11, 12) (NHM): petiole of areolet approximately 0.5× as long as 2rs-m; area superomedia rough, costula connecting at its anterior 0.3; confluent areas superomedia and petiolaris distinctly longitudinally concave; second tergite as long as or slightly longer than apical width; posterior margins of tergites 6 and 7 truncate; hind femora and tibiae entirely reddish brown; pterostigma brown.

## Supplementary Material

XML Treatment for Campoplex

XML Treatment for Campoplex bazariae
